# Chronic Bronchitis

**Published:** 1890-12

**Authors:** 


CHRONIC BRONCHITIS. (Bartholow.)

| | | |
|---|---|---|
| ℞. | Ext. Eucalyptus, - - - | oz. j. |
| | Ammonii Chloride, (granular) - | dr. ij. |
| | Ext. Glycyrrhizæ, - - - | oz. ij. |
| | Syr. Tolutani, - - - | oz. iij. |

